# Probable brucellosis-associated myocarditis presenting as myocardial infarction with non-obstructive coronary arteries: a case report

**DOI:** 10.1128/asmcr.00140-26

**Published:** 2026-10-13

**Authors:** Sameer Alzaidi, Maha Alhamdi, Areej Zubaid, Abdullah Altahini, Abdulaziz Alshalan, Ola Qadi, Ahmed Abdullah, Nawaf Alrabaie, Waad Alotaibi

**Affiliations:** 1 Division of Infectious Diseases, King Abdulaziz Specialist Hospital, Taif Health Cluster, Taif, Saudi Arabia; Vanderbilt University Medical Center, Nashville, Tennessee, USA

**Keywords:** brucellosis, myocarditis, MINOCA, *Brucella *spp.

## Abstract

**Background:**

Brucellosis remains an important zoonotic disease in endemic regions and has heterogeneous clinical presentations. Cardiovascular involvement is uncommon, with myocarditis representing a rare but potentially serious manifestation. Brucellosis-associated myocarditis may mimic acute coronary syndrome (ACS), creating diagnostic uncertainty when obstructive coronary artery disease is absent.

**Case Summary:**

We report a case of probable brucellosis-associated myocarditis in a 27-year-old previously healthy man with recently diagnosed brucellosis who developed acute chest pain and dyspnea accompanied by ischemic electrocardiographic changes and marked troponin elevation. Transthoracic echocardiography showed preserved biventricular systolic function without regional wall-motion abnormalities, significant valvular disease, or pericardial effusion. Coronary angiography demonstrated normal coronary arteries without obstructive disease, establishing a working diagnosis of myocardial infarction with non-obstructive coronary arteries (MINOCA). In the absence of cardiac magnetic resonance imaging (MRI) or endomyocardial biopsy, the suspected myocarditis etiology was supported by the temporal relationship with active brucellosis, dynamic biomarkers, and electrocardiographic changes, exclusion of obstructive coronary artery disease, and subsequent clinical recovery. Because antimicrobial and anti-inflammatory therapies were administered concurrently, the response cannot be attributed to either intervention alone. No relapse or recurrent cardiac symptoms occurred during six months of follow-up.

**Conclusion:**

In endemic regions, brucellosis-associated myocarditis should be considered among infectious causes of myocardial injury with non-obstructive coronary arteries. MINOCA is a working syndrome rather than an etiologic diagnosis. When cardiac MRI or endomyocardial biopsy is unavailable, epidemiologic exposure, microbiologic data, serial biomarkers, ECG evolution, echocardiography, and coronary angiography may support a probable diagnosis while acknowledging residual uncertainty.

## INTRODUCTION

*Brucella* species are facultative intracellular gram-negative coccobacilli transmitted primarily through ingestion of unpasteurized dairy products or direct contact with infected animals (1). Brucellosis remains endemic in several regions worldwide, particularly in the Middle East, the Mediterranean basin, and parts of Asia and Latin America (1, 2). Although the disease commonly presents with constitutional symptoms, cardiovascular involvement is rare, occurring in approximately 1%–2% of cases. Infective endocarditis represents the most common cardiac manifestation (1, 3). Myocarditis has primarily been reported in isolated case reports and small case series (4). We report a case of probable brucellosis-associated myocarditis that initially fulfilled a working clinical syndrome of myocardial infarction with non-obstructive coronary arteries (MINOCA). MINOCA is used here as a descriptive syndrome after angiography excluded obstructive coronary artery disease, not as the final etiologic diagnosis. We also summarize the diagnostic limitations encountered without cardiac magnetic resonance imaging or endomyocardial biopsy and compare the case with published reports of *Brucella*-associated myocarditis.

## CASE PRESENTATION

A 27-year-old Middle Eastern man presented on day 0 with 1 week of intermittent fever, fatigue, and lower back pain without cardiopulmonary symptoms. He had no significant medical history and reported recent ingestion of unpasteurized sheep cheese, although the interval between exposure and illness was uncertain. Temperature was 39.2°C, heart rate 111 beats/min, blood pressure 99/58 mmHg, respiratory rate 20 breaths/min, and oxygen saturation 95% on room air. Cardiac and pulmonary examinations were unremarkable, with no murmur, gallop, edema, or organomegaly.

Initial testing showed C-reactive protein 2.2 mg/dL, troponin I 9.04 µg/L, and platelets 128 × 10⁹/L; available liver tests and remaining hemogram parameters were within reference ranges (Table 1). Blood cultures showed no growth. External serum agglutination testing (SAT) for brucellosis was positive at >1:640; species-specific titers at our institution were lower (*B. melitensis* 1:80 and *B. abortus* 1:60). *Brucella* PCR was not performed. Compatible constitutional illness, unpasteurized dairy exposure, and serology supported a clinical diagnosis of brucellosis. No focal osteoarticular diagnosis was documented. He was discharged on doxycycline 100 mg twice daily and rifampicin 600 mg daily.

**TABLE 1:** Laboratory findings at admission and discharge

Variable (unit)	Admission	Discharge	Reference range
Platelet count (×10⁹/L)	128	202	150–410
Aspartate aminotransferase, AST (U/L)	29	18	11–34
Alanine aminotransferase, ALT (U/L)	20	15	0–40
C-reactive protein, CRP (mg/dL)	2.2	0.08	<0.5
Troponin I, TnI (µg/L)	9.04	0.01	<0.04

On day 1, he returned with acute substernal burning chest pain and dyspnea. Blood pressure was 100/62 mmHg and heart rate was 102 beats/min. Electrocardiography demonstrated ST-segment depression in the inferior leads (II, III, and aVF) and lateral precordial leads (V4–V6), with associated T-wave inversion (Fig. 1). Cardiac biomarkers were markedly elevated, with a dynamic troponin pattern (Fig. 2). Echocardiography demonstrated preserved biventricular systolic function without regional wall-motion abnormalities, significant valvular disease, or pericardial effusion. He was initially managed for possible acute coronary syndrome (ACS), but coronary angiography showed normal coronary arteries without obstruction, fulfilling a working MINOCA syndrome. In the context of recent brucellosis and acute myocardial injury, *Brucella*-associated myocarditis became the leading presumptive etiology. Cardiac magnetic resonance imaging (MRI) and endomyocardial biopsy were not obtained; therefore, the diagnosis remained probable rather than definitive.

**Fig 1:**
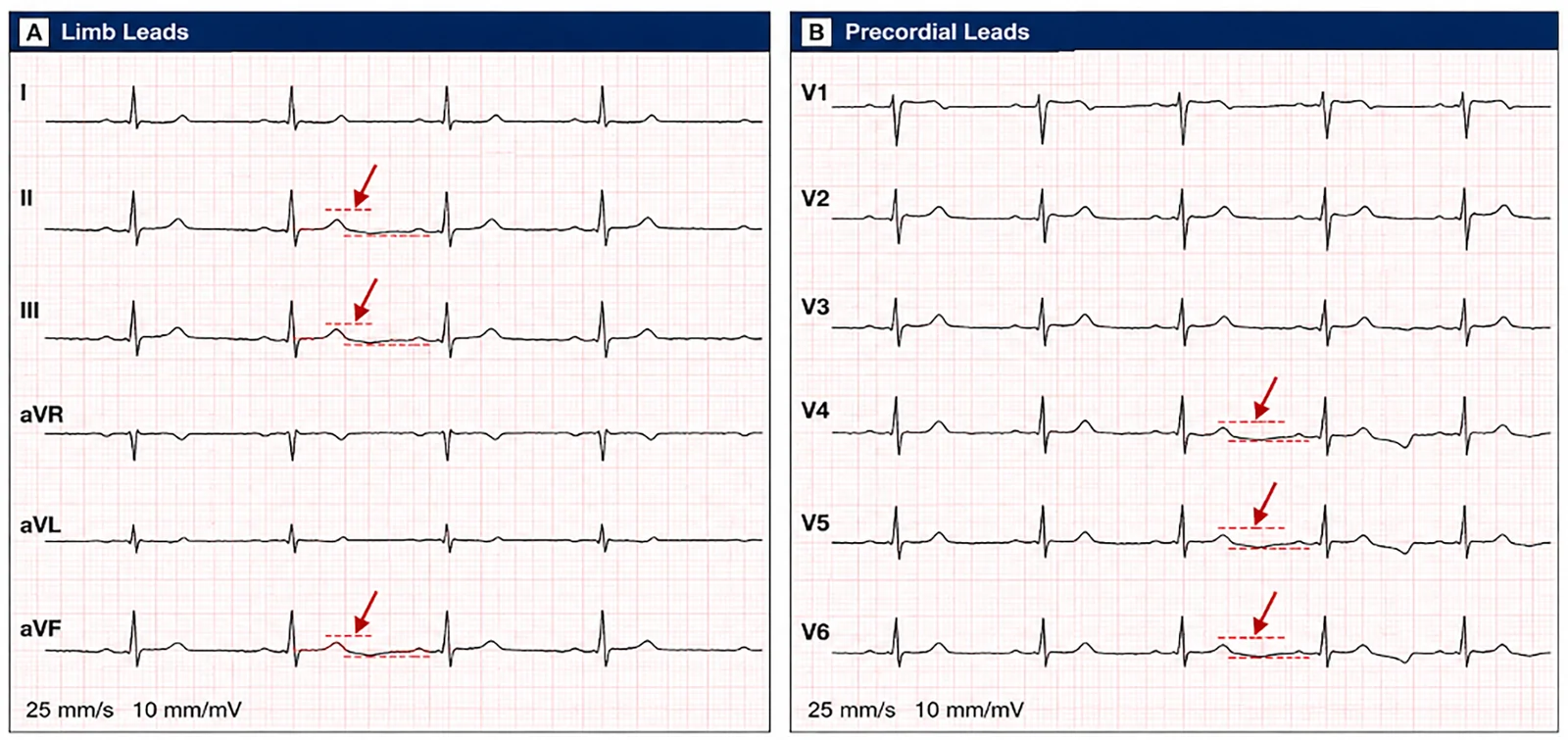
Twelve-lead electrocardiogram on admission. (**A**) Limb leads demonstrate ST-segment depression in the inferior leads (II, III, and aVF). (**B**) Precordial leads demonstrate ST-segment depression in the lateral precordial leads (V4–V6), accompanied by T-wave inversion. Red arrows indicate the ST-segment abnormalities. These findings were observed in the setting of acute chest pain and elevated cardiac troponin I. Subsequent coronary angiography showed no obstructive coronary artery disease.

**Fig 2:**
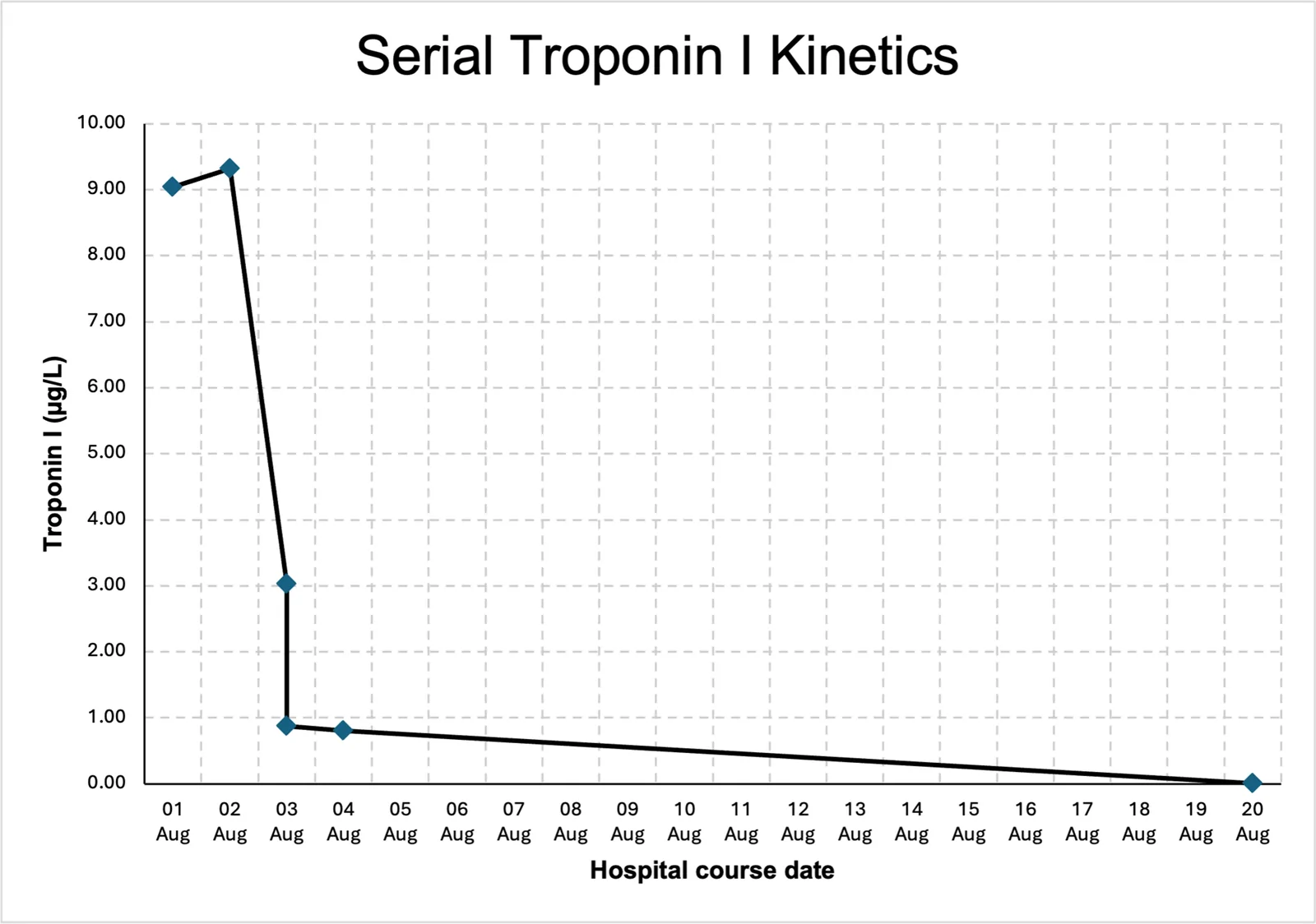
Serial cardiac troponin I kinetics. Serial cardiac troponin I measurements demonstrating markedly elevated at presentation, followed by a further increase to a peak, a rapid decline, and subsequent normalization. The graph illustrates the temporal changes in myocardial injury biomarkers throughout the clinical course.

Therapy was intensified to doxycycline 100 mg twice daily, rifampicin 900 mg daily, and intravenous gentamicin 320 mg (5 mg/kg) daily. Ibuprofen 600 mg every 8 h for 15 days and colchicine 0.5 mg twice daily for 3 months were added empirically for inflammatory chest pain. Definite pericarditis was not established. Troponin I fell to 0.88 µg/L by day 2, repeat echocardiography remained normal, and fever and chest pain resolved by day 4. No arrhythmias were observed. Because antimicrobial and anti-inflammatory therapies were administered concurrently, improvement cannot be attributed to either intervention alone.

The patient left against medical advice on day 5 after counseling. Outpatient therapy included doxycycline 100 mg twice daily, rifampicin 900 mg daily, and intramuscular streptomycin 1,000 mg daily to complete a 21-day aminoglycoside course, with ibuprofen and colchicine continued as planned. Follow-up showed sustained clinical recovery without antimicrobial toxicity (Table 1). After 21 days, streptomycin was stopped; doxycycline plus rifampicin continued for a total of 6 months. Ventricular function remained preserved, with no heart failure, relapse, or recurrent cardiac symptoms through approximately six months.

## DISCUSSION

Brucellosis remains endemic in the eastern Mediterranean region, including Saudi Arabia, where transmission is primarily associated with consumption of unpasteurized dairy products and contact with infected animals (1, 5–7). Although cardiovascular involvement occurs in only 1%–2% of cases, it accounts for a disproportionate share of brucellosis-related mortality. Infective endocarditis (IE) is the predominant cardiac manifestation, whereas isolated myocarditis without valvular involvement is exceedingly rare and has been described mainly in isolated case reports and small case series (1, 3–6, 8).

The differential diagnosis included infectious or autoimmune myocarditis, ACS, Takotsubo syndrome, infective endocarditis, and non-atherosclerotic mechanisms of MINOCA, including vasospasm, microvascular dysfunction, and thromboembolism. Angiography excluded obstructive epicardial coronary disease, and the absence of regional wall-motion abnormalities made Takotsubo syndrome less likely. Blood cultures were negative, and transthoracic echocardiography showed no vegetation or significant valvular abnormality; however, transesophageal echocardiography was not performed, so endocarditis cannot be excluded with certainty. Other infectious and autoimmune causes also remained possible because comprehensive virologic testing, autoimmune evaluation, cardiac MRI, and endomyocardial biopsy were not performed. Nevertheless, unpasteurized sheep-cheese exposure, preceding febrile illness, positive *Brucella* serology, dynamic troponin elevation, normal coronary angiography, and the clinical course collectively supported probable brucellosis-associated myocarditis as the leading diagnosis (1, 3, 4).

The apparent discrepancy between the initial *Brucella* serologic titer obtained at the referring institution (>1:640 by SAT) and the lower species-specific titers measured at our institution as shown in Table 2 (*B. melitensis* 1:80 and *B. abortus* 1:60) should not be interpreted as a simple biologic fall in antibody concentration. Agglutination assays use serial dilutions, and the endpoint is the highest dilution at which agglutination remains detectable. Reported titers can therefore vary according to assay format, antigen preparation, dilution scheme, endpoint interpretation, and laboratory-specific procedures. In addition, a composite *Brucella* agglutination result is not necessarily directly comparable with separately reported species-specific titers. Inter-laboratory variability in *Brucella* serology has been documented, and SAT results must be interpreted together with the clinical and epidemiologic context rather than in isolation (9, 10). This is particularly relevant in endemic settings, where background antibodies from prior exposure and limitations in sensitivity and specificity complicate interpretation (9, 11). Thus, the discordant titers in this case cannot establish either microbiologic progression or treatment response. The diagnosis rested on the totality of exposure history, compatible illness, serologic evidence, cardiac investigations, and longitudinal clinical course.

**TABLE 2:** *Brucella* serological findings at admission and discharge

Serological test	Admission	Discharge	Reference range
Brucella total titer	1:640	Not measured	<1:160
*Brucella melitensis*	1:80	1:40	<1:160
*Brucella abortus*	1:160	1:40	<1:160

Acute myocarditis may clinically mimic ACS because ECG abnormalities and cardiac biomarker elevation are nonspecific (12–14). As summarized in Table 3, reported *Brucella*-associated myocarditis cases commonly describe constitutional symptoms followed by chest pain, troponin elevation, and ischemia-like ECG changes (1, 3, 4, 15–17). The published cases summarized in the literature review also predominantly involved young adult men. This observation is consistent with the recognized male predominance of clinically apparent myocarditis in broader myocarditis populations, but the very small number of reported brucellosis cases precludes conclusions about a brucellosis-specific sex association. Our patient similarly developed myocardial injury after a systemic febrile illness. Importantly, the angiographic finding of non-obstructive coronary arteries defined the initial MINOCA syndrome, whereas suspected myocarditis represented the proposed underlying etiology; these terms are therefore not interchangeable. Unlike several previously reported cases supported by cardiac MRI, our diagnosis remained probable and relied on integration of epidemiologic, serologic, ECG, biomarker, echocardiographic, and angiographic findings (3, 15, 17).

**TABLE 3:** Published case reports of *Brucella*-associated myocarditis^*a*^

Study	Patient age (yr)/sex	Presentation	Cardiac findings	Diagnosis	Treatment	Outcome
Lagadinou et al. (1)	21/M	Fever, fatigue, retrosternal chest pain	ST elevation; MRI edema/fibrosis	Blood cultures + serology	Gentamicin + doxycycline/rifampin	No follow-up reported
Salehi et al. (3)	35/F	Palpitations, fever, fatigue	Mild LV dysfunction; MRI inflammation	Positive *Brucella* serology	Gentamicin + doxycycline/rifampin	MRI improved; residual fibrosis at 8 months
Gatselis et al. (4)	17/M	Chest pain, fever, night sweats	T-wave inversion; mild LV abnormalities	Blood cultures + serology	Streptomycin + doxycycline/rifampin	Normal ECG/ECHO at 12 months
Abid et al. (18)	32/M	Chest pain and fever	Normal ECHO; MRI delayed enhancement	Blood cultures + serology	Streptomycin + doxycycline/rifampin	No relapse at 3 months
Wendt et al. (15)	27/M	Retrosternal pain, diaphoresis, fever	MRI edema and delayed enhancement	Blood cultures + serology	Gentamicin + doxycycline/rifampin	Complete recovery
Khorasani et al. (16)	22/M	Dyspnea, pleuritic pain	Pericardial effusion; EF 45%	Blood cultures + serology	TMP-SMX + rifampin/doxycycline	Normalized LV function at 2 months
He et al. (17)	35/F	Dyspnea, cough, chest pain	ECG abnormalities; normal systolic function	Serology + BAL NGS	Doxycycline/rifampin + TMP-SMX/gentamicin	Complete recovery at 6 months

^*a*^ M, male; F, female; BAL, bronchoalveolar lavage; NGS, next-generation sequencing; SAT, serum agglutination test; EF, ejection fraction; LV, left ventricular; ECG, electrocardiogram; ECHO, echocardiography; MRI, magnetic resonance imaging; SAT, serum agglutination test; TMP-SMX, trimethoprim-sulfamethoxazole.

Another notable finding was the marked troponin elevation despite preserved ventricular systolic function and the absence of regional wall-motion abnormalities. Troponin indicates myocardial injury but does not identify its mechanism (12, 14). The rapid biomarker decline is compatible with a transient myocardial injury pattern, but it is not specific for inflammatory myocarditis and should not be interpreted as proof of mechanism. Preserved echocardiographic function likewise does not exclude myocarditis. Without cardiac MRI or endomyocardial biopsy, the extent and mechanism of myocardial injury could not be directly characterized.

Management of *Brucella*-associated myocarditis is not standardized and is largely derived from case reports and expert opinion (1, 3, 4, 15–18). In this patient, therapy was intensified to doxycycline, rifampicin, and an aminoglycoside after cardiac involvement was recognized. Rifampicin was increased from 600 to 900 mg daily. Although higher rifampicin doses have demonstrated increased pharmacokinetic exposure and encouraging efficacy in uncomplicated brucellosis, the contribution of dose escalation to this patient’s outcome cannot be determined from a single case (19). Ibuprofen and colchicine were used empirically for inflammatory chest pain, not as treatment of the underlying brucellosis infection. Because these agents were administered alongside intensified antimicrobial therapy, the subsequent clinical and biochemical improvement cannot establish which component produced the observed response, and improvement should not be presented as evidence of direct antimicrobial causality.

This case emphasizes three points: brucellosis-associated myocarditis should remain in the differential diagnosis of myocardial injury in endemic settings; MINOCA is a working syndrome that requires investigation of the underlying mechanism; and when cardiac MRI, endomyocardial biopsy, transesophageal echocardiography, or comprehensive infectious and autoimmune testing is unavailable, diagnostic certainty is necessarily limited. In that setting, exposure history, microbiologic evidence, serial biomarkers, ECG evolution, echocardiography, and angiography can support a probable diagnosis when competing etiologies and limitations are explicitly acknowledged (1, 3, 14).

### Conclusion

Brucellosis-associated myocarditis is an uncommon but recognized complication that may mimic ACS and present initially as MINOCA. In endemic regions, brucellosis infection should be considered when myocardial injury occurs with non-obstructive coronary arteries and compatible epidemiologic features. Because cardiac MRI and endomyocardial biopsy were not obtained, this case is best classified as probable brucellosis-associated myocarditis. Distinguishing the MINOCA syndrome from its underlying etiology and integrating exposure, microbiologic, biomarker, ECG, echocardiographic, and angiographic evidence are essential when definitive testing is unavailable.
